# Bilateral, Simultaneous Medial Meniscus Bucket Handle Tears in a 23-Year-Old Female

**DOI:** 10.1155/2014/689130

**Published:** 2014-09-11

**Authors:** Brett Walker, Andrew Limbert

**Affiliations:** Department of Orthopedic Surgery, Michigan State University, McLaren Oakland, Pontiac, MI 48342, USA

## Abstract

Traumatic tears of the meniscus are well reported in the literature. Rarely, bilateral meniscal tears occur. A PubMed search found that only three cases of bilateral medial meniscus bucket handle type tears have been reported. Treatment options range from partial meniscectomy to repair of the meniscal tear. Repair is reported to be more successful in the vascular red-red or red-white zones. We present the case of a 23-year-old female who sustained simultaneous bilateral medial meniscus bucket handle type tears in an automobile accident. She was treated in a staged fashion with knee arthroscopy. Her meniscus tears were both found to be in the vascularized zone and meniscal tear repair was conducted. At two weeks postoperatively, she had excellent resolution of her symptoms and has returned to pain-free weight-bearing. She has remained pain-free at six-month follow-up.

## 1. Introduction

Traumatic meniscal tears are well reported. They are responsible for significant pain and loss of function. Infrequently, traumatic meniscal tears are bilateral. After a PubMed literature search, only three reports of bilateral traumatic bucket handle meniscus tears have been reported to our knowledge. None of these cases appeared to occur in the red-red or red-white zone bilaterally. The following case is a 23-year-old female who was involved in a motor vehicle collision where she suffered simultaneous bilateral bucket handle type meniscus tears, which were both located in the red-white zone. Neither was associated with ACL pathology. To our knowledge, this is the first example in the literature.

## 2. Case

A 23-year-old female was seen in the emergency department regarding bilateral knee pain after a motor vehicle collision. As she was restrained, she likely experienced a sudden flexion force across the knees. Plain film radiography demonstrated no abnormalities and she was discharged home. On follow-up, she complained of mild swelling with clicking of her knees which caused her pain. She denied any locking sensation of her knees. Prior to the accident, she had no complaints of knee pain.

Plain film radiography was repeated in office and demonstrated no pathology.

The patient's physical exam revealed no visual deformity. She ambulated with a slightly antalgic gait. There were mild effusions present bilaterally. Passive range of motion was limited secondary to pain. There was a five-degree extension lag present on the right knee while the left knee achieved full extension. Flexion was symmetric to 135 degrees with pain at the extreme. She complained of pain with medial joint line compression and McMurray's testing was positive medially (both audible clicking and pain) bilaterally. Lachman test was stable and symmetric bilaterally. There was no posterior lag. Valgus and varus stressing was normal. Further clinical knee testing was negative. She admitted that her right knee was more symptomatic than the left one.

MRI revealed findings consistent with bilateral medial meniscus bucket handle type tears. There was no evidence of abnormal meniscal shape. All ligaments, including her ACLs, were intact bilaterally. No further pathology was noted.

**Figure 1:**
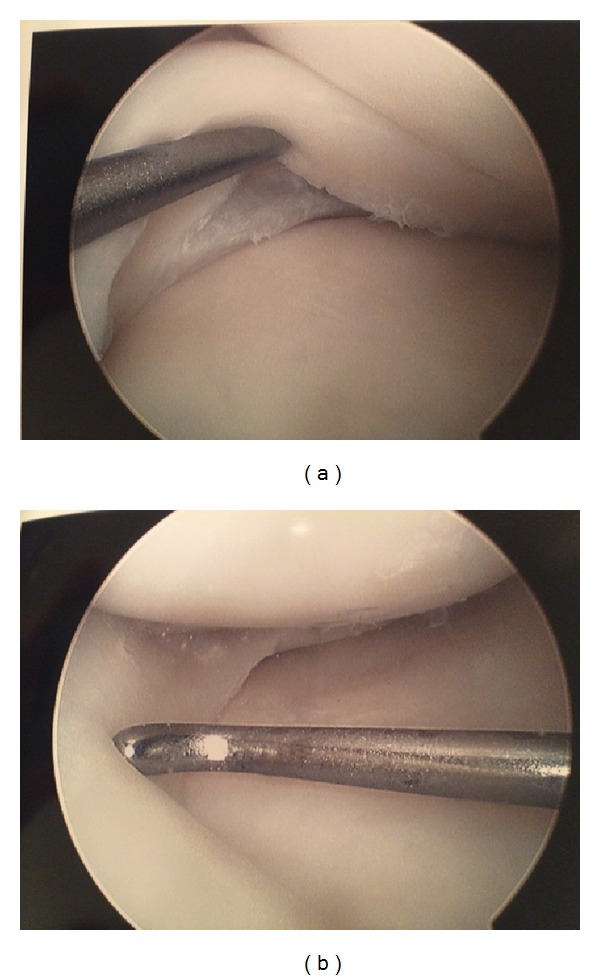
(a) Left knee, prefixation. (b) Left knee, postfixation.

Attention was paid to the right knee first, as the patient had limited passive range of motion and was where she reported the most pain. Traditional anteromedial and anterolateral portals were utilized. On initial visualization, no tear was noted of the medial meniscus. When the probe was introduced, however, there was a clear medial meniscus bucket handle type tear. The tear appeared in the red-white zone of the meniscus as the posterior aspect of the tear appeared pink and was assumed to be within the vascularized zone. The edges of the tear did not appear to be frayed. Based on these observations, the decision was made to repair the meniscus rather than resect it. An all inside knotless suture-anchor technique was used. Three anchors were placed and the integrity of the meniscus was tested using the probe. This provided excellent stabilization of the tear. Further examination of the remainder of the knee revealed no further pathology (Figure 1).

**Figure 2:**
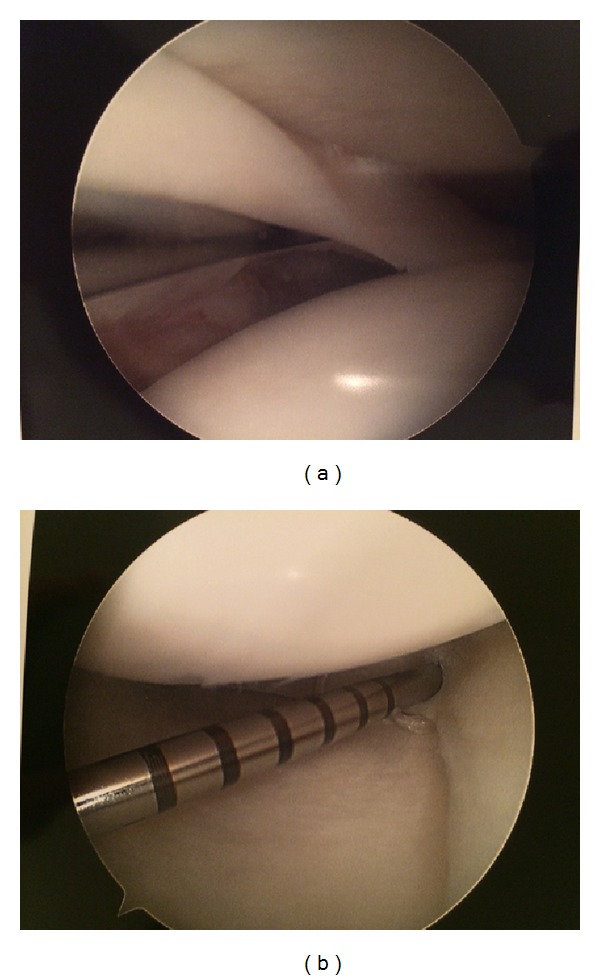
(a) Right knee, prefixation. (b) Right knee, postfixation.

Four weeks later, the patient returned to the operative suite to have the left knee addressed. Once again, traditional anteromedial and anterolateral portals were utilized. A nondisplaced bucket handle type tear was again observed in the red-white zone of the medial meniscus. Probe examination revealed that the edges were not frayed. In similar fashion as the right knee, the meniscus was repaired. Two anchors were placed and probe examination revealed excellent stabilization of the tear (Figure 2).

Patient was followed closely postoperatively for each knee. She reported near total resolution of her knee pain at her first postoperative visit at two weeks. On subsequent follow-up, her knee pain had totally resolved and she was weight bearing without difficulty or pain. She has remained symptom-free at six months. Informed consent was obtained for participation in this case study.

## 3. Discussion

The above case highlights traumatic bilateral medial menisci bucket handle tears. No previously reported cases of these repairs were found on a PubMed literature search. Our patient achieved excellent results and is pain-free after bilateral repair.

Bucket handle tears are most frequently caused by simple twisting injury usually related to sport. Shakespeare and Rigby reported that out of 196 patients only 4 (2%) were sustained from road traffic accidents [1]. Bucket handle tears are thought to represent 10–26% of all meniscus tears with most cases reported unilateral and often associated with ACL pathology. Of the cases which are bilateral, most occur nonsimultaneously [2].

Blood supply to the meniscus is from branches of the medial and lateral inferior and middle geniculate arteries [3]. The outer 25–30% of the meniscus has sufficient blood supply to heal. This represents the “red-red” zone and partially the “red-white” zone [4]. Previous reports have shown higher failure rates for isolated medial meniscus tears without ligamentous pathology [2]. This may occur because of intrinsic factors. It has been reported that although medial tears do not heal as well as lateral tears, the majority of meniscal repairs will go on to healing and patients report satisfactory results at up to five-year follow-up. Even elite athletes may return to previous levels of play [5].

During a literature search, three cases were found of bilateral medial meniscus bucket handle tears. Day et al. reported a 35-year-old male who sustained bilateral medial meniscus bucket handle tears [3]. These, however, occurred nonsimultaneously and were attributed to a history of many football injuries. Greis and Lahav also reported nonsimultaneously occurring bucket handle type medial meniscus tears in a female ice skater [6]. The closest example to our case was presented by Abbott et al. who described simultaneously occurring bilateral bucket handle tears in a long jumper [7]. None of these cases appeared to be amenable to repair bilaterally.

Our case appears to be unique to those previously found in the literature. It represents simultaneous, bilaterally occurring bucket handle style meniscus tears found in the periphery secondary to motor vehicle trauma, which is a rare mechanism of injury.
